# Editorial: Evolving social hierarchies: current patterns of stratification in Latin America and the Caribbean

**DOI:** 10.3389/fsoc.2026.2013173

**Published:** 2026-10-20

**Authors:** Vicente Espinoza, Graziela Serroni Perosa

**Affiliations:** 1 School of Social Sciences, Universidad de la República, Montevideo, Uruguay; 2 School of Arts, Sciences, and Humanities, Universidade de Sao Paulo, São Paulo, Brazil

**Keywords:** educational attainment, intersectionality, Latin America & Caribbean, redistributive preferences, social inequalities, social mobility

The Research Topic offers an up-to-date compilation of contemporary scholarship on social inequality in the region. The Research Topic comprises 18 articles authored by scholars from eight countries across Latin America and beyond, reflecting the growing vitality of stratification research in the region. The contributions cover a wide range of national settings as well as comparative cross-national analyses.

Latin American and Caribbean cases can illuminate the analysis in contemporary societies where mounting inequality is an inescapable fact (Piketty, 2013; Stiglitz, 2013). New insights could be gained from the analyses presented here about the effects of inequality on the social order. Latin America and the Caribbean are particularly suited for the understanding of the structural effects of economic inequality as this has been a long-term trait that survived from colonial times in spite of modernization, pervading many domains of social life (Hoffman and Centeno, 2003; Torche, 2014).

Papers in this Research Topic have a common concern about the mechanisms that engender, reproduce and legitimate unequal life chances, understanding social stratification as a multidimensional and dynamic process. To a large extent this view of inequality connects classical mobility research focused on the interaction between social origins and destinations, net of structural processes, with newer approaches centered on recognition, intersectionality, and the legitimacy of inequality.

Unlike classical Latin American and Caribbean studies of class and inequality, these papers focus explicitly on identifying the specific processes that produce inequality. Mobility studies in the region during the 1960s compared with those of the last two decades show contrasting profiles. During the 20th century, social mobility was mostly associated with large-scale structural changes, such as demographic transition, rural-urban migration, industrialization and urbanization.

Social development as well as dependency scholars expected that industrialization and urbanization, along with modern value orientations supported by redistributive public policies would bring about a general improvement in life conditions. Since the 1970's market reforms and military rule, that put an end to redistribution policies, shifting the focus of inequality studies to the analysis of dismal living conditions expressed in poverty and informal labor markets. Additionally, poverty studies brought into focus processes and groups somehow disregarded in previous analyses, notably the gender component of inequality, as well as its ethnic, territorial, or migratory dimensions.

The contributions in this Research Topic uncover specific mechanisms of inequality rather than elaborating on major questions about capitalism, modernization or social order. They present research on how educational institutions, social relations and ascriptive characteristics affect people's opportunities and shape their beliefs and evaluations. Shared concerns about interrelated dimensions of inequality allow diverse analytical traditions to converge on a common object of inquiry without requiring adherence to a single overarching theoretical framework. Rather than falling into a single category, the papers in this Research Topic form a highly interconnected thematic network. This is characteristic of the intellectual field in the region where scholars also elaborate on the implications of their research for other areas of inequality.

The articles in this Research Topic address a problematic situation in the modernization of Latin American and Caribbean societies: the process of intergenerational mobility driven by educational attainment, supported by public policies enhancing human capital did not prevent the reproduction of social inequality. In this context, they conceive of social inequality as an outcome of structural limitations of social inclusion models, and consider these limitations in four main fields: social mobility, education, the legitimation of the social order, and intersectional approaches to class.

Social mobility and class structure are the backbone of the studies on social stratification as they test the distribution of opportunities across social groups. The studies in this research offer a counterpoint to contemporary approaches to social mobility based on log-linear and log-multiplicative analyses of the mobility table (Breen, 2004; Erikson and Goldthorpe, 1992; Hout, 2007; Solís and Boado, 2016). In this vein Ricardi Morgavi offers a comparative examination of social mobility by cohorts in Latin American and European countries. The concern for the changes in class structure is also present, especially in the paper by Boado et al. that provides a historical view for Uruguay to assess hypotheses about the levels of mobility. Other studies of social mobility link the process to aspects of social structure that strengthen the explanation of occupational attainment, notably social networks structures. Nieto-Rivera compares the reproduction of disadvantages across generations in Chile and Mexico, linking them to education, social capital and the vulnerability of social positions. The paper by Otero et al. studies how short-term social mobility associates with the diversity of social contacts showing a limited permeabilility of class boundaries. Dalle and Carrascosa delve in the complexity of social mobility by linking it to micro-level processes across generations, ethnic descent, territories and occupational networks. Finally, Chávez Molina and Rodríguez de la Fuente focus on automation through artificial intelligence addressing the heterogeneity it introduces in class positions and living conditions.

In the classic model of status attainment (Blau and Duncan, 1967) education is the driving force of upward mobility by weakening the association with social origin (ascription) and also by returns to educational achievement. Five papers in Research Topic examine the extent to which education had helped to moderate inequality in Latin America. Boniolo and Lemos analyze educational transitions from primary to higher education comparing Argentina, Chile, and Mexico. They show that, despite the expansion of access at all educational levels, the reduction of educational and social inequalities was uneven and limited, as social origin continued to shape educational attainment. Ziegler and Giovine examine the current operation of Argentina's egalitarian model of access to education to show that its equalizing contribution has been challenged by institutional factors such as hidden selectivity mechanisms and the expansion of private education. Reverón Peña offers an encompassing explanation of the linkages from educational transitions to social inequality, considering class, social capital and territorial segregation. On socioeconomic factors affecting education achievement see Cabra Hernández. Two papers focus on tertiary education. Alves de Brito shows how quota policies governing the access to higher education widen opportunity structures for the disadvantaged. Tabilo-Prieto analyses how the first generation with college education make sense of this experience, showing that initial feelings of inadequacy give way to an appreciation of diversity, coupled with a critical view of social injustices.

Social inequality describes the distribution of some valued resource in society. A set of papers in Research Topic extend the subject to the perception of fairness in the distribution, examining to what extent and on what grounds do people justify inequalities. Meritocratic beliefs—defined as the distribution of social rewards on the basis of individual effort—serve as a key justification for social justice, addressing the difficulties of predicting preferences for redistribution from class positions. Three papers on Research Topic place their focus on the legitimacy of inequality in Chile, where social inequalities persist despite personal effort. Castillo et al. investigate why the consistent support for an income-based pension system cuts across social classes to find that meritocratic beliefs explain it better. Iturra-Sanhueza et al. show a positive association between subjective social status and meritocratic perceptions somehow modified by income and educational levels. Biehl et al. use a life-history survey to test the association between employment trajectories and distribution preferences finding that a history of unstable employment correlates with lower support for universal policies.

Three papers examine the relationship of gender and race/ethnicity with class, challenging the alleged gender or race neutrality of class studies, offering an analysis of the interplay of these dimensions at different levels. Ney Simons et al. provide an intersectional analysis of social class in Honduras by examining the interaction among several dimensions of inequality: gender, ethnicity, urbanization, sector of activity, and labor relations. Muñiz Terra et al. compare the interplay of class and gender in the life-histories of women under dissimilar welfare systems in Argentina and Cuba, finding convergences in the patterns of labor-market participation. Castillo and Salgado show that ethno-racial configurations expressed in skin color have a significant effect on educational attainment.

In summary, this Research Topic provides a comprehensive overview of current debates and research agendas on inequality in Latin America and the Caribbean. Ultimately, it shows how structural features of social inequality in Latin America are actively reproduced by institutional and ideological processes. By shifting analytical focus from macro-level trends to micro-level mechanisms—such as hidden educational selectivity, social network closure, and meritocratic legitimation—the volume uncovers why intergenerational mobility remains bottlenecked despite human capital expansion. It reveals a central theoretical paradox: modern social relations often coexist with, and are cushioned by, subterranean exclusionary practices and ingrained normative beliefs. Consequently, these 18 studies challenge regional stratification research with research outcomes that capture how structural advantage, policy friction, and intersectional power dynamics jointly perpetuate enduring social hierarchies.
